# Silibinin prevents depression-like behaviors in a single prolonged stress rat model: the possible role of serotonin

**DOI:** 10.1186/s12906-020-2868-y

**Published:** 2020-03-06

**Authors:** Bombi Lee, Gwang Muk Choi, Bongjun Sur

**Affiliations:** 1 Acupuncture and Meridian Science Research Center, College of Korean Medicine, Kyung Hee University, 26, Kyungheedae-ro, Dongdaemun-gu, Seoul, 02447 Republic of Korea; 2 Center for Converging Humanities, Kyung Hee University, 02447 Seoul, Republic of Korea; 3 The Graduate School of Basic Science of Medicine, College of Medicine, Kyung Hee University, 02447 Seoul, Republic of Korea; 4 Department of Molecular medicine and TIDRC, School of Medicine, Ewha Womans University, 07985 Seoul, Republic of Korea

**Keywords:** Silibinin, Post-traumatic stress disorder, Depression, Single prolonged stress, Serotonin

## Abstract

**Background:**

Post-traumatic stress disorder (PTSD) is an extreme mood disorder that occurs after experiencing extreme stress, and patients with this disorder are known to accompany with symptoms of depression, anxiety, and memory impairments. Silibinin (SIL) is a natural polyphenolic flavonoid and is the main active ingredient of silymarin, which is primarily extracted from the milk thistle. Although some studies have assessed the properties of this flavonoid, the potential of SIL as a treatment for PTSD patients and its mechanisms of action have yet to be fully elucidated.

**Methods:**

After exposure to a model of single prolonged stress (SPS), the open field test (OFT) and forced swimming test (FST), were used to investigate the effects of SIL on anxiety- and depression-like symptoms in male rats. The rats received of SIL (25, 50, and 100 mg/kg) for 14 days following exposure to SPS.

**Results:**

Administration of SIL significantly improved anxiety-like behaviors in the OFT, depression-like behaviors in the FST, and freezing behavior in fear conditioning test. SIL also increased levels of serotonin in the hippocampus (Hipp) and amygdala, and enhanced expression of tryptophan hydroxylase-1 mRNA in the Hipp. The administration of SIL also inhibited SPS-induced decreases dopamine levels and increases norepinephrine levels in the Hipp.

**Conclusions:**

Taken together, the present findings suggest that SIL can be a useful therapeutic ingredient for the treatment of trauma stress-associated symptoms, including PTSD-induced anxiety and depression caused by PTSD.

## Background

Post-traumatic stress disorder (PTSD) is a serious mental illness that manifests as a psychological reaction following the experience of a life-threatening situation and intense stress [1]. This mental disorder is caused by the traumatic stress induced by witnessing a shocking or fearful event; most of these traumas occur suddenly, cause severe distress in the person experiencing it, and overwhelm that individual’s general ability to cope with stress [2]. Furthermore, the stressful conflicts are re-experienced in conjunction with current events and conditioned stimuli can continue to produce fear responses that cause significant issues. The biological factors underlying PTSD include a variety of neurotransmitters (e.g. dopamine [DA] and norepinephrine [NE], and serotonin [5-HT]), are related to benzodiazepine receptors, and involve function within the hypothalamus-pituitary-adrenal (HPA) axis.

Of the several animal models of PTSD, the single prolonged stress (SPS) is the most common [3]. Mice that experience SPS exhibit anxiety- and depression-like behaviors and also show general and fear-related memory disorders [3], which is similar to the pattern of clinical symptoms observed in PTSD patients [4]. Physiologically, PTSD alters the neurochemical balances and changes the structure of the brain [5]. For example, PTSD increases hippocampal loss, which is associated with alterations in associative learning and spatial memory, and enhances amygdala (Amg) activation, which contributes to the commitment of traumatic events into long-term memory [5]. Accordingly, mice that experience PTSD like symptoms following repeated exposure to traumatic events exhibit the overgeneralization of fear in an environmental context. These changes may be due to imbalances in monoamine levels within certain regions of the neural fear circuit, such as the hippocampus (Hipp), Amg, and prefrontal cortex (PFC) [4]. Furthermore, dysregulation within the HPA axis, stress-induced cellular damage, and neuronal remodeling contribute to the occurrence of PTSD [6] and cause alterations in mental behaviors that are expressive of anxiety- and depression-like symptoms [6].

In recent studies of PTSD-mediated psychiatric conditions, the primary focus has shifted to the neurobiology underlying these disorders and the different neurotransmitter systems that are affected, including the serotoninergic system [1]. The depletion of 5-HT may be an important risk factor for the manifestations of anxiety- and depression-like symptoms in PTSD. In fact, some studies suggested that decreases of 5-HT in the Hipp and Amg precipitate the relapse of depression [7] and that alterations in 5-HT levels, which occur in association with PTSD and SPS, contribute to several PTSD-related behaviors, including anxiety, depression and fear memory [7]. Thus, the statuses of the serotonergic system and its related 5-HT, amino acids (e.g., tryptophan [TRP]), and enzymes (tryptophan hydroxylase [TPH]) are closely intimately involved in the pathology of PTSD [8].

Selective serotonin reuptake inhibitors (SSRIs) are the most commonly used to treat for depression, anxiety, and insomnia [9]. Although SSRIs is an effective cure for many patients with PTSD, the side effects associated with its long terms use make it disadvantageous [10]. Thus, it is necessary to develop novel therapeutic drugs for the treatment of PTSD and to clarify the effects of natural drugs that many be safer for its long-term treatment [11]. Sibibinin (SIL) is a natural polyphenolic flavonoid that is the main active ingredient in silymarin, which is mainly extracted from the milk thistle. SIL has traditionally been used to treat liver disease [12] and is known to exert antioxidative, anti-inflammatory, anti-cancer, cardioprotective, and neuroprotective effects in animal models of ischemia, Parkinson’s disease (PD), and Alzheimer’s disease (AD) [13]. The anti-inflammatory effects of SIL occur via the inhibition of nuclear factor-kappa B (NF-κB), a transcription factor that regulates gene expression related to neuroinflammation and the immune response [14]. The neuroprotective effects of SIL have been demonstrated against ethanol-induced brain damage as well as neurotoxicity caused by lipopolysaccharide (LPS) [15]. Additionally, SIL exerts anti-apoptotic, anti-inflammatory and neuroprotective effects in the 1-methyl-4-phenylpyridinium (MPP)-induced and 6-hydroxydopamine (6-OHDA)-induced animal models of PD [16, 17]. Other studies have shown that SIL enhances memory function in animals with amyloid β (Aβ)-induced memory deficits and after oxidative stress [18]. Furthermore, SIL protects the rat brain from methamphetamine-induced memory loss, and streptozotocin-induced cognitive deficits [19]. It has also been shown that SIL, including silymarin, reverses neurochemical and biochemical alterations in the Hipp that are induced by chronic unpredictable mild stress (CUMS) [20, 21], which suggests that SIL elicits significant antidepressant-like activities in the CUMS model of depression. SIL is thought to be the primary factor responsible for the pharmacological activities of silymarin; however, this notion has yet to be systematically corroborated and evidence is lacking regarding the efficacy of SIL versus that of silymarin. On the other hand, the abovementioned studies highlight the positive effects of SIL and its pharmacological activities, as well as its potential as a therapeutic component for the prevention or treatment of trauma stress-related psychological diseases, including PTSD. To date, no studies have determined whether administration of SIL can improve behavioral changes in a rat model of SPS-induced PTSD. Our research group investigating the neurobiology of anxiety- and depression-like behaviors in PTSD-related psychological conditions have focused on imbalances within the monoamine system, including 5-HT, DA and NE. Thus, in the present study, rats were exposed to SPS to induce an animal model of PTSD and the effects of SIL on the manifestation of PTSD-like behaviors were evaluated.

## Methods

### Animals and silibinin administration

The male Sprague-Dawley (SD) rats used in the experiment were 8 weeks old with a body weight of 220~350 g, and were purchased from Samtako Animal Co., Seoul, Korea. The animals were kept and given food and water freely in a sterile animal room with a light and dark cycle of 12:12 h. Animal experiments were conducted in accordance with the Code of Ethics and the Guidelines for the management of laboratory animals by the Animal Care and Use Committee of Kyung Hee University, which also approved the experimental protocol [KHUASP (SE)-15–115].

**Fig. 1:**
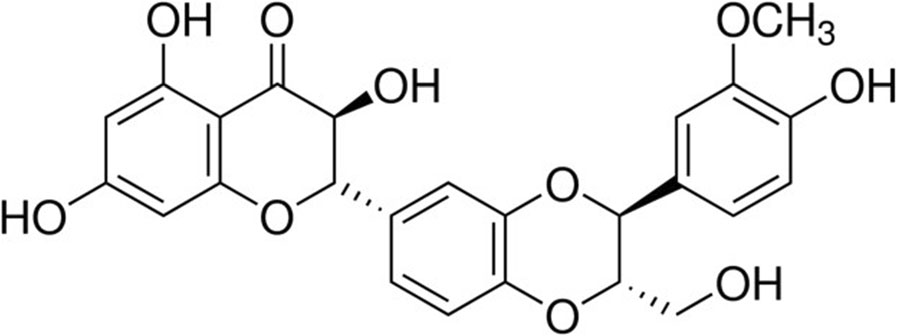
Chemical structure of SIL

**Fig. 2:**
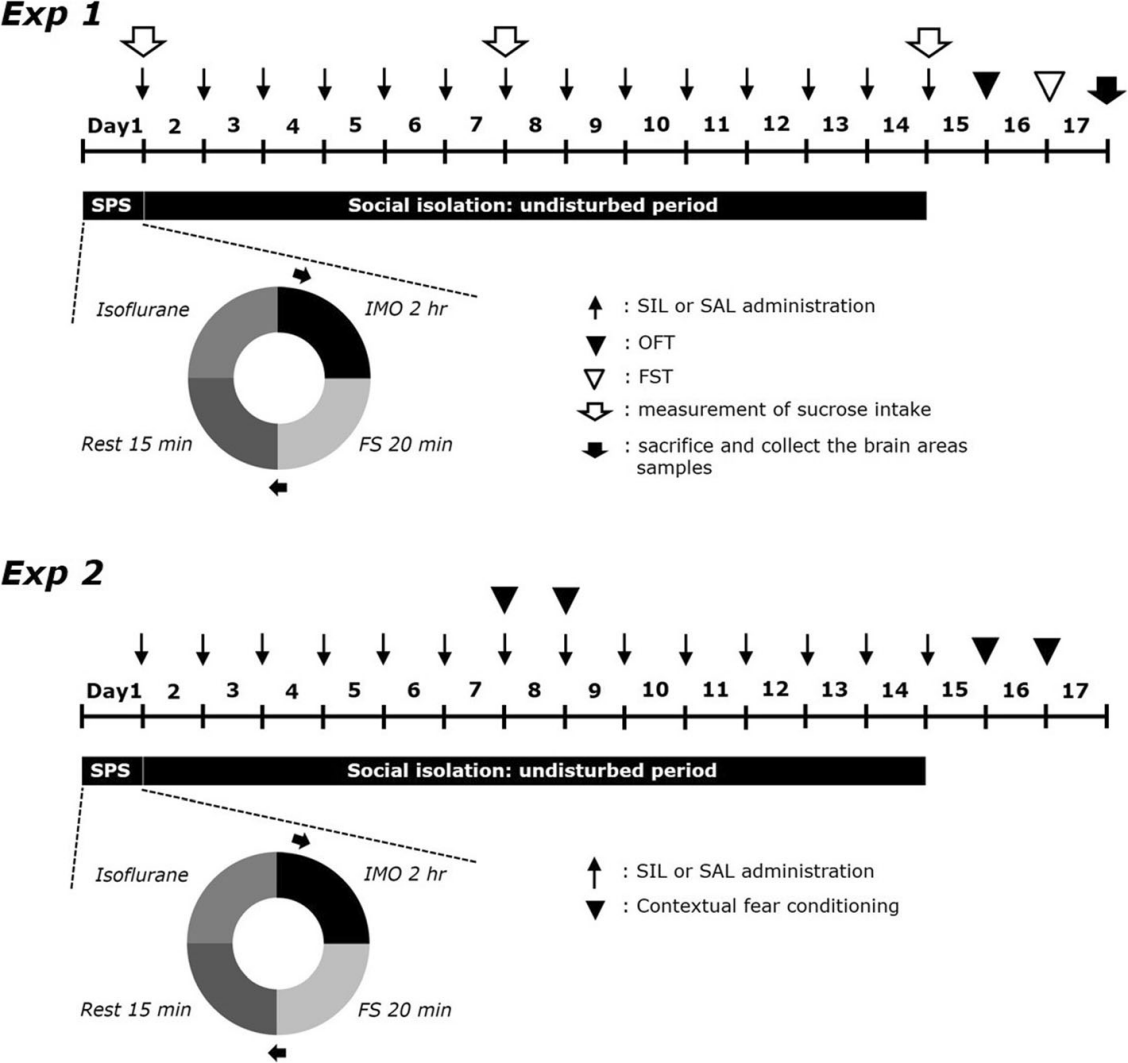
Experimental protocols for the induction of SPS-related depression-like behaviors and SIL treatment in rats. Different groups of rats (*n* = 7~8 per group) were used for each experimental condition

After exposure to SPS, SIL (25, 50, and 100 mg/kg, SIL; Sigma-Aldrich Chemical Co., St. Louise, MO, USA) and fluoxetine hydrochloride (10 mg/kg, FLX, Sigma) were intraperitoneally injected daily for 14 days. The control group without SPS exposure and SPS-induced only rats were intraperitoneally administered with saline at the same volume. The chemical structure of SIL is shown in Fig. 1. SIL and FLX were dissolved in 0.9% saline before i.p. administration. The body weight of the rats were also measured daily for 14 days. The overall experimental schedules of all drug administrations and behavioral tests are shown in Fig. 2.

Rats were divided at random into SAL-treated control group (SAL group, *n* = 7), SPS-induced rats treated with saline (SPS group, *n* = 8), SPS-induced rats treated with 25 mg/kg of SIL (SPS + SIL25 group, *n* = 7), SPS-induced rats treated with 50 mg/kg of SIL (SPS + SIL50 group, n = 7), SPS-induced rats treated with 100 mg/kg of SIL (SPS + SIL100 group, n = 7) and SPS-induced rats treated with 10 mg/kg of FLX (SPS + FLX group, n = 7).

### Single prolonged stress

This experimental method was performed to express symptoms similar to PTSD in an animal model. Briefly, the rats were immobilized using the same device as the restraint stress device for 2 h, and were subjected to forced swimming in the tank for 20 min. In addition, the rats that had been forced to swim were restored to their original conditions for 15 min. The rats were then exposed to isoflurane until rat was unconscious. To further enhance the mental extremes of PTSD, rats were left in their cages for 14 days without anyone’s obstruction [22].

### Behavioral test

#### Forced swimming test

The forced swimming test (FST) was measured as described previously [23]. In a transparent Plexiglas cylinder (20 cm diameter × 50 cm height) filled to a depth of 30 cm with 25 °C water, rats were put in the tank. The first 2 min of the total 6 min were not measured as it was the adaption time. The immobility time of the rats for the last 4 min were measured. Immobility is defined the instance where the head of the rat is above the water level and there is only minimal movement from the rat in order for it to expose only its head.

#### Measurement of sucrose intake

Sucrose intake was measured as described previously [24]. After the SPS, the confirmation of the unpleasant response to the stimulus, other than the depression-related behavioral changes, was determined through the change in the intake of 1% sucrose solution of the experimental animals. Before confirming the change in the sucrose solution intake, the rats were allowed to ingest the sucrose solution 24 h before the test in order to adapt the rats to the 1% sucrose solution. Six hours before the test, water and food were removed, and the change in sucrose solution intake for three hours on the day of the test was confirmed by the change in the weight of the water bottle. The measurement was performed three times: before stress-induction (1 day), and at the 1st and 2nd weeks after stress induction.

#### Open field test

Before completion of the FST trial, rats were exposed to the open field test (OFT) as previously described [25]. To determine the difference in activity between SPS exposure and each drug treatment, various behaviors in the open field were observed. The quadrangle container (60 × 60 × 30 cm) used for the experiment is a box made of wood, and the bottom is divided into a checkerboard-shaped section made of horizontal and vertical lines spaced 15 cm apart and the standard is divided into a central area and a peripheral area. Rats exhibit exploratory behavior when exposed to a new environment and can walk through the indicators. Also, rats that prefer darker locations tend to move more along the walled perimeter than in the open central areas. Emotion can be assessed by measuring the distance around the central area and the surrounding area. The locomotor activity was measured total distance and speed of movements traveled in the area. The total number of line crossings and the number of grooming were recorded in 5 min. The OFT is equipped with an incandescent lamp (60 W, 220 V) in the ceiling. Locomotor activities were monitored by a computerized video-tracking system using the S-MART Tracker Ver. 1.3 (PanLab Co., Barcelona, Spain).

#### Contextual fear conditioning test

Rats were subjected to contextual fear conditioning and extinction following SPS procedure. The contextual fear conditioning tests were performed as previously described [24]. Animals were condition to a sound while inducing shock. In order to activate the fear memory in the course of the experiment, the rats were placed in a container and a tone was played without the consequence of a shock. Rats were exposed for 5 min on days 7 and 14. Behavior was chosen because it was previously shown to induce a re-experiencing of the aversive incident and to promote behavioral sensitization. The rate of freezing is divided by the total time.

### Corticosterone, tryptophan, serotonin, norepinephrine and dopamine measurements

Following the 14 days period designated to allow the development of PTSD, corticosterone (CORT) levels in the plasma, 5-HT level in the Hipp, PFC, striatum (STR), and Amg, and NE, DA, TRP, and 5-hydroxyindoleacetic acid (5-HIAA) levels in the Hipp were measured as described previously [24]. Each group (*n* = 4/group) were anesthetized through inhalation of isoflurane (Hanlim Pharm. Seoul, Korea)(6%).

Plasma was collected via the abdominal aorta (*n* = 7~8 per group), after which the Hipp, PFC, STR, and Amg were removed from the brain. CORT, 5-HT, NE, DA, TRP, and 5-HIAA concentrations were evaluated by competitive enzyme-linked immunoassays (ELISAs) using antibodies against CORT (Novus Biologicals, LLC., Littleton, CO, USA), 5-HT (Abcam, Cambridge, UK), NE (Novus Biologicals), DA (Abcam), 5-HIAA (Abcam) and TRP (Biocompare, San Francisco, CA, USA).

### Total RNA preparation and reverse transcription-polymerase chain reaction

Expression of tryptophan hydroxylase-1 (TPH-1) and tyrosine hydroxylase (TH) mRNA was measured. In order to analyze TPH-1 and TH mRNA expression levels, mRNA was isolated from brain of rat using Trizol (Invitrogen Corp., CA, USA), and cDNA was synthesized using reverse transcription-polymerase chain reaction (RT-PCR) [24]. To extract RNA, total RNA was isolated from the Hipp of each rat. Quantification of RNA was diluted 50-fold and the absorbance was measured at 260 nm using a UV/vis spectrophotometer. After RT-PCR premix (Bioneer, Korea), sense primer, antisense primer (Bioneer), RNA, and DEPC treated distilled water were added to make a 50 μl final volume. Then RT-PCR was performed. cDNA was then amplified by PCR at 60 °C for 28 cycles to produce TPH-1 and 58 °C for 28 cycles to produce TH using Taq DNA polymerase (Takara) on a thermal cycler (MJ Research, Watertown, MA, USA). After synthesizing cDNA by RT-PCR method, it was observed in UV by electrophoresis at 50 V for 40 min using 1% agarose gel containing 0.5 μg/ml ethidium bromide (EtBr; Sigma). PCR cycle numbers for each gene were sufficient for the production of TPH-1 and TH. Data were normalized against GADPH expression in the corresponding sample.

### Statistical analysis

SPSS 13.0 (Chicago, IL, USA) was used for all statistical analyses, and the data were expressed as mean ± SEM. The data were analyzed using multi-way analysis of variance (ANOVA) and Tukey’s post hoc tests. Statistical significance at *p-value* < 0.05 has been assigned symbols in each figure.

## Results

### Effects of silibinin on SPS-induced body weight and plasma corticosterone levels

**Fig. 3:**
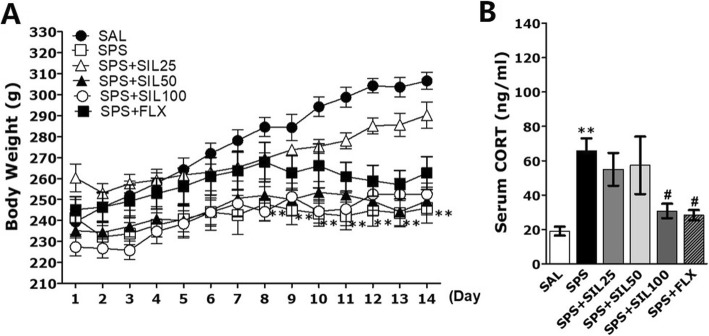
Effects of SIL on body weight (**a**) and plasma CORT levels (**b**) in rats exposed to SPS. Body weight was significantly lower in SPS-exposed rats than in saline-treated (SAL) rats (significant main effect of SPS exposure vs. control handling; n = 7~8/group). ^**^p < 0.01 vs. SAL group; ^#^*p* < 0.05 vs. SPS group

After SPS-induction, the body weight of each rat was measured daily for 14 days. Rats exposed to SPS began to lose body weight starting from day 1. This stress-induced decrease in body weight lasted several days. In some of the rats, there was a gradual gain in body weight but in some cases the weight did not return to the normal body weight (Fig. 3a). One-way ANOVA revealed significant differences among experimental groups: time effect [F (13,416) = 48.725, *p* < 0.01], group effect [F (5,32) = 4.425, *p* < 0.05], and group and time interaction effect [F (65,416) = 5.410, *p* < 0.01]. At the end 2 weeks, the rats in the SPS-only group had a body weight that was significantly lower than that that of the saline-treated (SAL) group [SPS group (F (2,23) = 15.23; day 1 versus day 14, *p* < 0.05]. The body weight of rats in the SPS group started to significantly decrease from day 8 as compared to that of the SAL group (*p* < 0.01). However, this loss of body weight was attenuated in SPS rats treated with 25, 50, or 100 mg/kg of SIL compared to that of the SPS group, but it was not statistically significant.

Also, it showed that SPS rats appeared significantly increased plasma CORT levels (346.05%) compared to the SAL group (*p* < 0.01; Fig. 3b) using ELISA analysis. These results may display that the SPS procedure is extremely stressful and the stress caused by this procedure could cause physiological symptoms of PTSD. However, administration of SIL has been shown to significantly inhibit the SPS-induced increase in plasma CORT levels (*p* < 0.05).

### Effects of silibinin on SPS-induced depression-like behavior

**Fig. 4:**
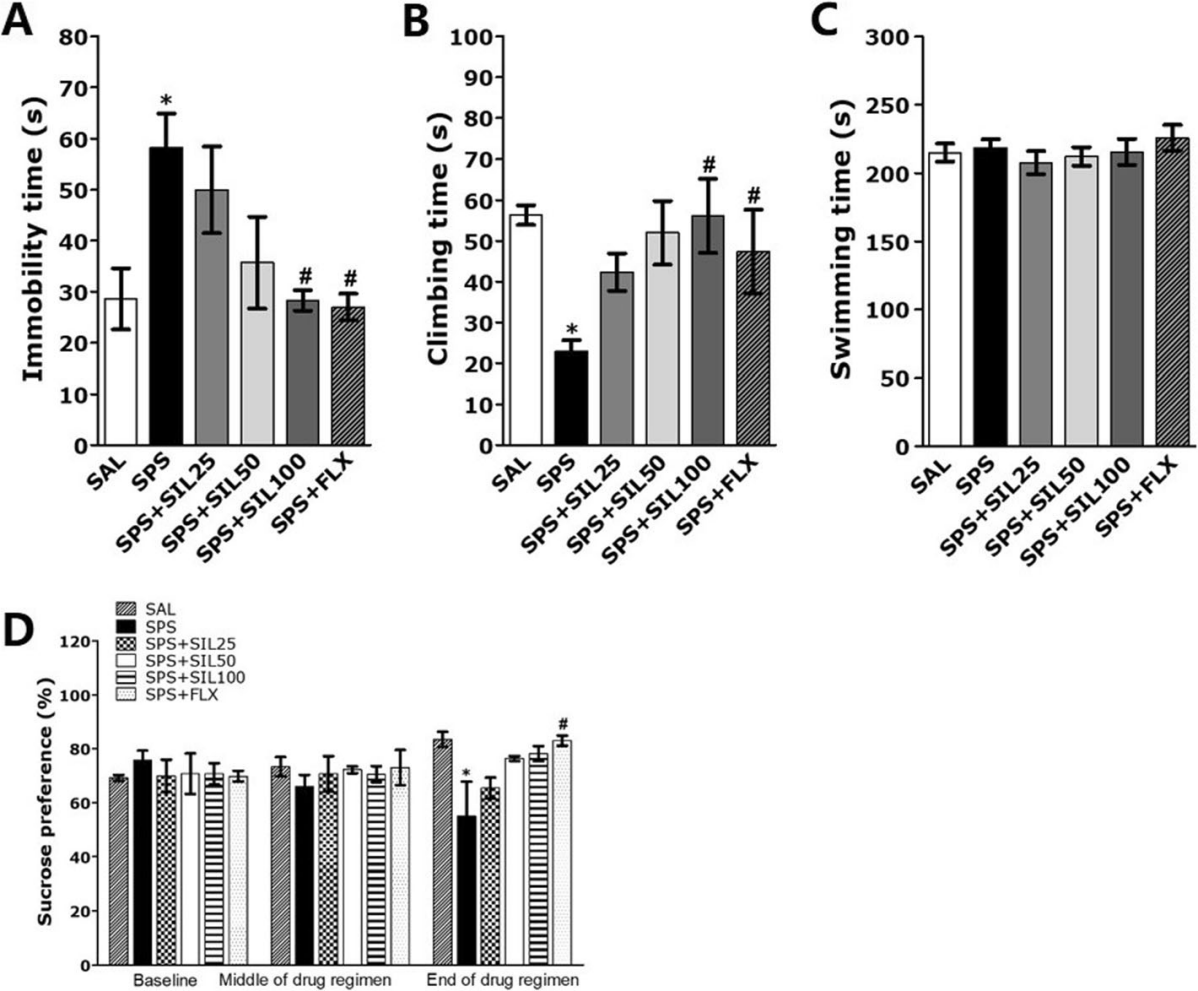
Effects of SIL on immobility time (**a**), climbing behavior (**b**), and swimming time (**c**) in the FST and sucrose intake (**d**) following exposure to SPS. ^*^p < 0.05 vs. SAL group; ^#^*p* < 0.05 vs. SPS group

The anti-depressive effect of SIL was evaluated in rats exposed to SPS by measuring the immobility time during the FST (Fig. 4a). A significant effect of SIL administration was observed, and administration of SIL significantly reduced the immobility time in the FST (*p* < 0.05). These results showed a significant reduction in the manifestations of depression-like behavior. Similarly, SIL administration showed a significant increase in the climbing time in the FST (*p* < 0.05, Fig. 4b). This shows that the significant effects on the immobility and climbing behaviors of the rats showed that SIL administration can significantly reduce depression-like behaviors. FLX also showed significant effects on immobility and climbing behavior in the FST. However, there was no significant differences in swimming behaviors between groups during the FST (*p* = 0.557; Fig. 4c).

Anhedonia, a symptom of depression, was measured through the monitoring of sucrose preference. Sucrose intake was measured 1 day before SPS exposure, 7 days and 14 days after SPS exposure. Analysis of sucrose preference disclosed that it was significantly decreased in the SPS group at day 14 compared to the SAL group (*p* < 0.05; Fig. 4d). However, the sucrose intake of rats treated with SIL (100 mg/kg) was increased compared to the SPS group, but it was not statistically significant.

### Effects of silibinin on SPS-induced anxiety-like behavior

**Fig. 5:**
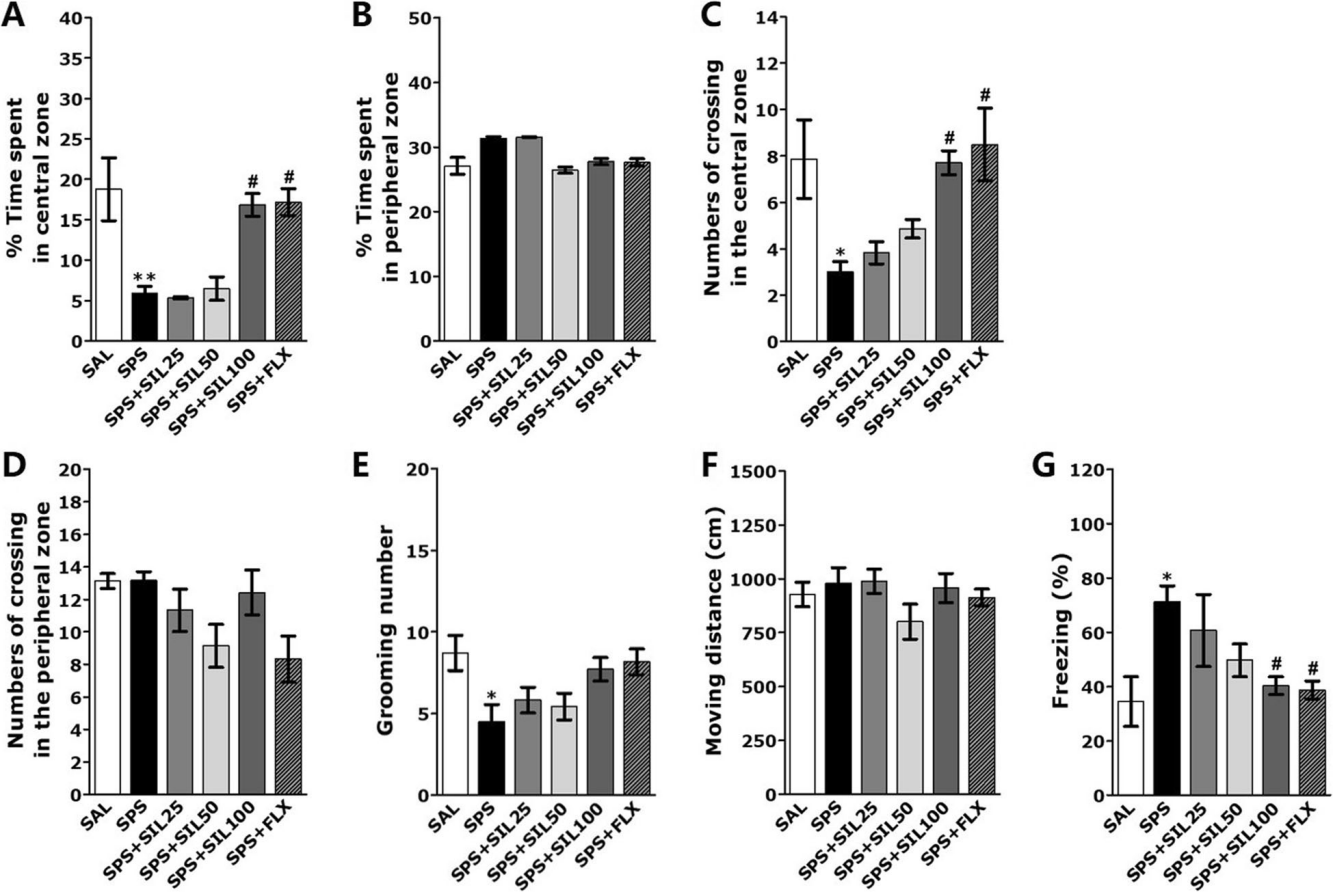
Effects of SIL on locomotion in the OFT in rats exposed to SPS. Changes in time spent in the central zone (**a**) and peripheral zone (**b**), the number of lines crossed in the central zone (**c**), and peripheral zone (**d**), the number of grooming bouts (**e**), locomotor activity measured by total moving distance (**f**), and freezing behavior (**g**). ^*^p < 0.05, ^**^p < 0.01 vs. SAL group; ^#^*p* < 0.05 vs. SPS group

Rats exposed to SPS spent significantly less time in the central zone (CZ) compared to the SAL group (*p* < 0.01; Fig. 5a). In addition, rats exposed to SPS showed a significant decrease in the number of CZ crossings in the CZ compared to the SAL group during the OFT (*p* < 0.05; Fig. 5c). The SPS group showed no significant differences in the time spent and the number of crossings in the peripheral zone (PZ) between groups (*p* = 0.673 and *p* = 0.712, respectively; Fig. 5b and d). However, rats treated with SIL at a dose of 100 mg/kg spent significantly more time in the CZ and showed a significant increase in the number of CZ crossings compared with the SPS group (*p* < 0.05, respectively). The anxiety-like behavior of the SPS + SIL100 group were similar to those of the SPS + FLX group. Grooming behavior, such as the washing of their snout and head, was mainly observed and measured in the rectangular container. The numbers of grooming bouts observed in the OFT (Fig. 5e). Alternations in grooming behaviors were increased in rats treated with SIL (100 mg/kg), but it was not statistically significant. Comparisons performed using a parametric one-way ANOVA also showed no significant differences among the groups in terms of locomotor activity in the OFT [F (5,37) = 0.202, *p* = 0.959](Fig. 5f).

### Effects of silibinin on SPS-induced contextual freezing behavior

We examined the rat’s situational freezing behavior after exposure to SPS. After exposure to SPS, the SPS group significantly had an increased freezing time, in comparison to the SAL group on day 14 (*p* < 0.05; Fig. 5g). However, in the group treated with SIL (100 mg/kg), the time required to demonstrate the freezing time was significantly reduced compared with the SPS group (*p* < 0.05). These results show that the persistent fear response to the original context was significantly related to early trauma and could be attenuated by SIL treatment.

### Effects of silibinin on SPS-induced 5-HT, NE, and DA levels in the brain

**Fig. 6:**
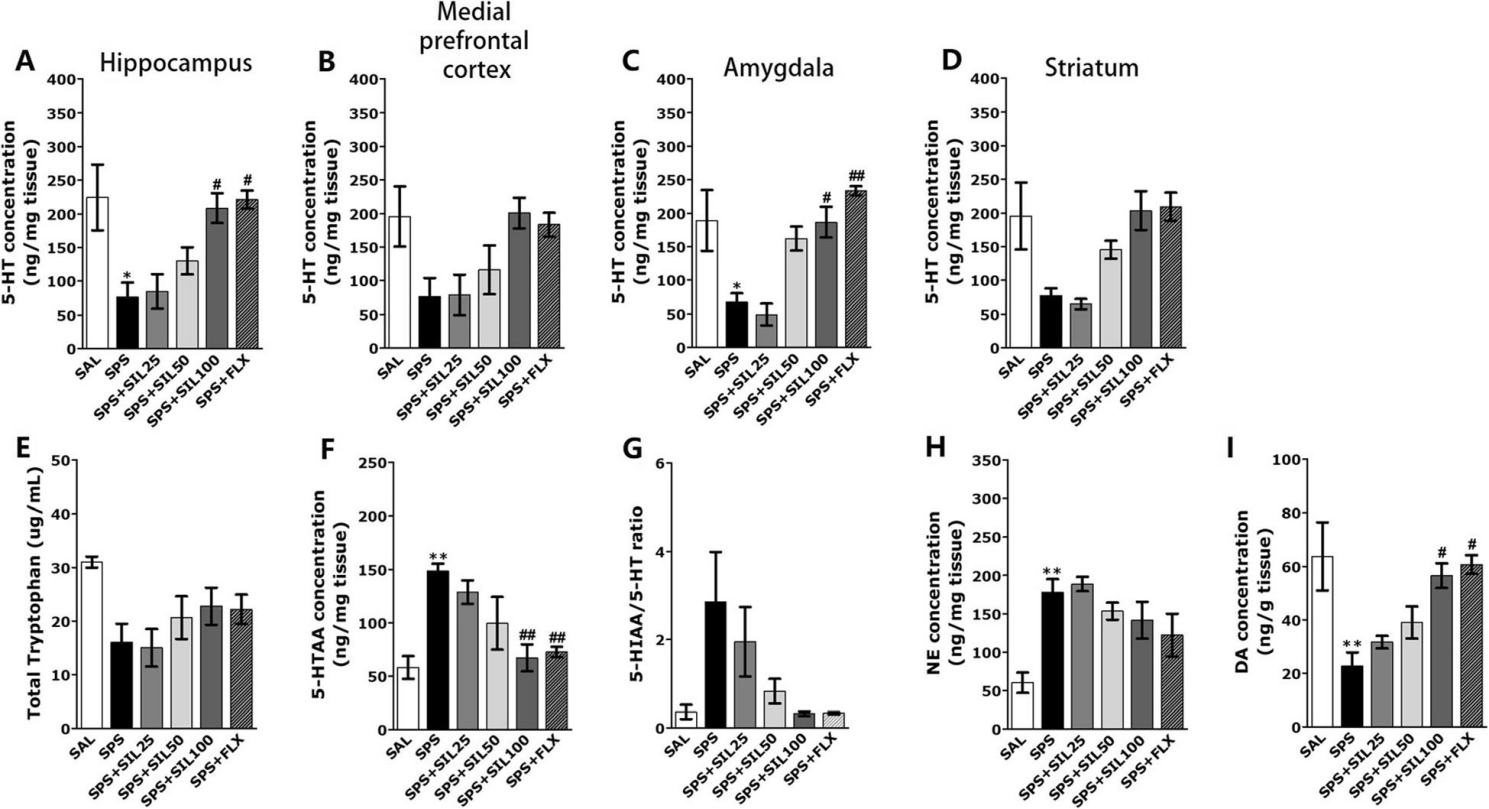
Effects of SIL on 5-HT concentrations in the Hipp (**a**), PFC (**b**), Amg (**c**), and STR (**d**); plasma TRP levels (**e**), 5-HIAA (**f**), and the 5-HIAA/5-HT ratio (**f**) in the Hipp; NE concentrations in the Hipp (**e**); and DA concentrations in the Hipp (**f**). ^*^p < 0.05, ^**^p < 0.01 vs. SAL group; ^#^*p* < 0.05, ^##^*p* < 0.01 vs. SPS group

Figure 6 shows the differences in the 5-HT levels in the Hipp, PFC, STR, and Amg among the groups. It showed a significant difference in the 5-HT levels in the Hipp of the rats [F (5,20) = 5.354, *p* < 0.05]. These results revealed that the SPS group had significantly lower 5-HT levels in the Hipp compared to the SAL group (*p* < 0.05; Fig. 6a). However, in the group treated with SIL (100 mg/kg), the reduction of 5-HT level in the Hipp was significantly inhibited compared to the SPS group (*p* < 0.05). Although these results exhibited that the levels of 5-HT of SPS group in the PFC was decreased compared to the SAL group, it was not statistically significant (*p* = 0.119; Fig. 6b). In addition, the *post-hoc* test result showed that the levels of 5-HT of SPS group in the STR were relatively decreased compared to the SAL group, but it was not statistically significant (*p* = 0.247; Fig. 6d).

Not only that, these results showed that the level of 5-HT of the SPS group in the Amg was significantly lower than that of the SAL group (Fig. 6c). However, in the group treated with SIL (100 mg/kg), the reduction of the dose of 5-HT in the Amg was significantly inhibited compared to the SPS group (*p* < 0.05). Furthermore, the 5-HT levels in the Hipp and Amg of rats receiving of SIL (100 mg/kg) were similar to those of the rats receiving of FLX (10 mg/kg).

It showed that the SPS rats exhibited significantly decreased TRP (51.61 %) in the Hipp compared to the SAL group, but it was not statistically significant (*p*=0.95; Fig. 6e) using ELISA analysis. ELISA analysis discovered that SPS procedure resulted in increases in the 5-HIAA (256.03 %) levels in the Hipp compared to the 5-HIAA hippocampal concentration in rats in the SAL group (*p*<0.01; Fig. 6f). Administration of SIL (100 mg/kg) significantly inhibited these SPS-induced increases in the 5-HIAA levels in the Hipp (*p*<0.01). Chronic SIL treatment (100 mg/kg) decreased the 5-HIAA/5-HT ratio in the Hipp, but it was not statistically significant (Fig. 6g).With the use of ELISA, the NE levels were found to be significantly increased in the Hipp of SPS rats (296.67%) compared to the SAL group (*p* < 0.01; Fig. 6h). However, in the group treated with SIL (100 mg/kg), the increase of NE level in the Hipp was significantly inhibited compared to the SPS group, but it was not statistically significant.

More ELISA analysis revealed that SPS rats exhibited significantly decreased DA levels in the Hipp (35.69%) compared to the SAL group (*p* < 0.01; Fig. 6i). However, in the group treated with SIL (100 mg/kg), the decrease of DA in the Hipp was significantly inhibited compared to the SPS group (*p* < 0.05).

### Effects of silibinin on SPS-induced TPH-1 and TH mRNA in the Hipp

**Fig. 7:**
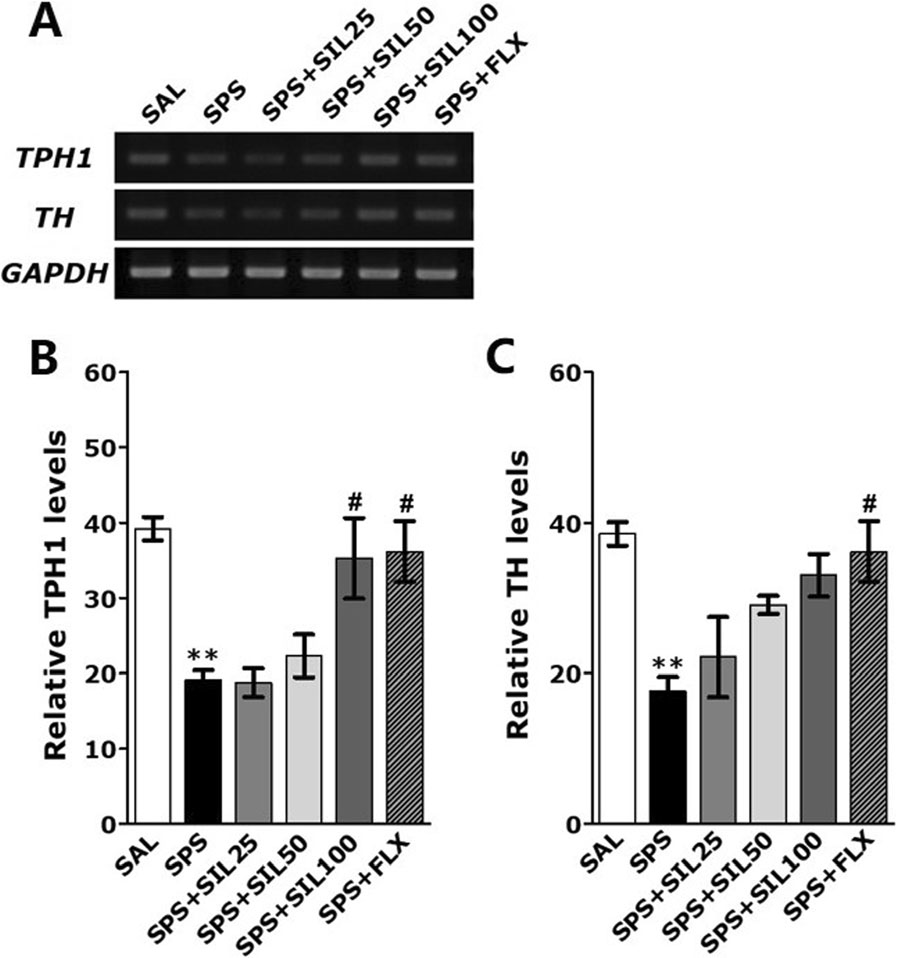
Effects of SIL on the mRNA expression TPH-1 and TH in the Hipp of rats exposed to SPS for 14 consecutive days. PCR bands on agarose gels and relative intensities are shown. ^**^p < 0.01 vs. SAL group; ^#^*p* < 0.05 vs. SPS group

To examine the effect of SIL treatment on the expression of TPH-1 and TH in the Hipp of rats impaired by SPS, the mRNA expression of TPH-1 and TH were analyzed using RT-PCR (Fig. 7). The expression of TPH-1 and TH in the Hipp showed a significant difference among the groups [F (5,23)=8.670, *p*<0.01 and F (5,23)=6.657, *p*<0.01]. In the Hipp, TPH-1 mRNA expression were significantly lower in the SPS group, compared to the SAL group (*p*<0.01). In the group treated with SIL (100 mg/kg), the decrease of TPH-1 mRNA expression in the Hipp was significantly inhibited compared to the SPS group (*p*<0.05). However, in the group treated with SIL (100 mg/kg), the decrease of TH mRNA expression in the Hipp was inhibited compared to the SPS group, but it was not statistically significant. In the Hipp of rats receiving of SIL (100 mg/kg), TPH-1 mRNA expression were similar to those of rats receiving of FLX (10 mg/kg).

## Discussion

The present results show that SPS increased the immobility time of rats in the FST, which is indicative of depression-like behavior. However, the administration of SIL significantly reduced immobility time during the FST and also significantly increased the amount of time spent at the center of the open field in the OFT. Additionally, the present results showed that SIL prevented decreases in 5-HT levels in the Hipp and Amg and recovered DA levels in the brain. Thus, the present findings support the anti-depressive and anxiolytic effects of SIL in a rat model of SPS-induced PTSD.

Of the various psychiatric diseases, PTSD is unique because its diagnosis requires exposure to a traumatic situation that precipitates, and thus precedes, the occurrence of behavioral symptoms [3]. Common PTSD symptoms include anxiety, substance abuse disorders, and major depression [3]. In the present study, a model of SPS-induced traumatic stress was employed due to its reliable recreation of numerous PTSD-like symptoms, including the manifestation of more anxiety- and depression-like behaviors in experimental animals relative to unstressed control animals, which is consistent with previous studies [26]. In the SPS model of depression, plasma CORT levels are significantly higher due to dysregulation of the HPA axis, which has been associated with anxiety- and depression-like behaviors [27]. This high concentration of plasma CORT indicates that rats in this model are subjected to extremely stressful situations [27]. Consistent with previous findings, the present study found that rats had lower body weights and higher plasma CORT levels after SPS. However, the administration of SIL inhibited these effects, which suggests that SIL reduced plasma CORT levels by modulating activity in the HPA axis to restore neurochemical and behavioral responses and to reduce the psychological effects associated with dysfunction in the HPA axis. Furthermore, the anti-stress actions of SIL indicate its potential as an antidepressant and anxiolytic medication.

Anhedonia, which is a decrease in the ability to experience pleasure, is a core symptom of depression in humans [28]. The sucrose preference test has revealed that exposure to chronic stress results in expression of the core and common endophenotypes of major depressive disorder, including avoidance, helplessness, and anhedonia [29]. 5-HT regulates reward processing and anhedonic behaviors. For example, chronic treatment with SSRIs reverses psychostimulant withdrawal-induced anhedonia in rats [30] and alters sucrose preference in mice [31]. Similarly, combined treatment with FLX and a 5-HT1A antagonist, which rapidly elevates 5-HT levels in forebrain structures, reverses psychostimulant withdrawal-induced anhedonia in rats [30]. Taken together, these findings indicate that SIL treatment rapidly elevates 5-HT levels in the Hipp and increases sucrose preference, which is indicative of the restoration of behavioral and neurochemical responses.

During the FST, most animals will try to escape by enthusiastically swimming. However, the unstable position induced in rodents by the FST is considered to be similar to a state of depression or helplessness in humans [32]. The present findings are consistent with those of previous studies showing that SPS increases immobility time during the FST [27]. In the present study, the administration of SIL significantly decreased immobility time and increased climbing time in the FST. However, there were no significant individual differences in swimming times among the groups, which suggests that SIL administration did not affect motor function. Some patients with depression also exhibit a rapid loss of body weight and a lack of pleasure. In the present study, exposure to SPS significantly decreased body weight and induced anhedonia, which is similar to previous animal experiments of SPS-related anxiety and depression. Although treatment with SIL reversed these changes, the effects were not significant. Regardless, the administration of SIL significantly restored serum CORT levels stressed rats compared to the saline-treated control group, which suggests that SIL inhibited PTSD-induced psychological dysfunction associated with the HPA axis.

Many studies have shown that depression-related symptoms and abnormal social behaviors are often accompanied by anxiety-related symptoms [32]. Additionally, rats exposed to unpredictable mild stress for 3-weeks exhibited various behavioral disturbances, including depression-like behaviors such as a decreased immobility time in the FST and fewer crossings in the OFT [33]. In the present study, rats exposed to SPS exhibited anxiety-like behaviors, as well as depression-like behaviors. However, the administration of SIL after exposure to SPS significantly increased time spent in the CZ and the number of lines crossed in the OFT. Our results suggest that SIL may decreased trauma stress-induced fear memory in the OFT. Furthermore, the present study revealed a reduced fear response in the contextual fear conditioning test.

The Hipp plays an important role in glucocorticoid-related negative feedback. For example, abnormal negative feedback has been observed in subjects with depression, anxiety, and PTSD and it is also likely that CORT and NE are involved in the pathophysiologies of these disorders [34]. Animal experiments have shown that there are chronic elevations in plasma CORT levels due to stress as well as after the application of CORT to damaged hippocampal neurons [34] and structural neuroimaging studies have revealed that the hippocampal formation has a reduced volume in PTSD patients. Therefore, hippocampal neurons, which play important roles in the manifestation of PTSD-like symptoms, are particularly vulnerable to PTSD-specific neuroendocrine abnormalieies [34].

Many studies have reported increased levels of NE and decreased levels of DA in the brains of PTSD patients, and that these changes are correlated with disease severity [35]. Other studies have reported that the pathophysiologies of PTSD-induced anxiety and depression are enhanced by imbalances in monoamines levels, including NE, DA and 5-HT [36]. Recently, a behavioral method for measuring individual response categories in the FST, including immobility, climbing, and swimming, was developed [27]. It is widely accepted that increased immobility time in the FST is related to decrease 5-HT levels in the Hipp and Amg whereas increased climbing behaviors are indicative of upregulated NE release [37]. All antidepressants reduce immobility time in the FST but, from a pharmacological standpoint, these drugs also induce at least two different behavioral patterns [26]. For example, SSRIs (e.g., FLX) reduce immobility behavior whereas drugs that predominantly cause an increase in the extracellular levels of NE or a decrease in the extracellular levels of DA may increase climbing behavior [36]. Additionally, withdrawal from chronic reserpine treatment significantly decreases climbing behavior in the FST and it is possible that these changes could be rescued by increased activation within the noradrenergic system [36]. The present study showed that SIL treatment inhibited SPS-induced increases in NE levels and decreases in DA levels in the Hipp, which indicates that SIL may indirectly affect activation of the noradrenergic system through the synthesis of DA and regulation of NE in the Hipp. Therefore, SIL can modulate the imbalances in monoamine levels that generally occur in the brain, especially in the Hipp, due to PTSD.

5-HT is centrally involved in the modulation of behavior and emotions. In the present study, SIL treatment inhibited SPS-induced decreases of 5-HT levels in the brain, which indicates that SIL indirectly altered the regulation of monoamine synthesis as well as that of 5-HT. Moreover, consistent with previous findings [1], the present study found that SIL treatment in rats exposed to SPS significantly increased 5-HT levels in the Hipp and Amg to near-baseline levels. This finding suggests that SIL may be able to alleviate anxiety- and depression-like behaviors through neurochemical changes via regulation of the serotonergic system in the brain [38]. Additionally, SIL had significant effects on 5-HT metabolites, including 5-HIAA. More specifically, the increased 5-HT levels and decreased the 5-HIAA/5-HT ratio following chronic SIL treatment were indicative of a change in serotonin metabolism. Thus, the present results suggest a basis for further examining the serotonergic mechanisms underlying the anti-depressant and anxiolytic effects of SIL.

Rats exposed to SPS also exhibit reductions in the mRNA expression of TPH-1 in the Hipp, which leads to anxiety- and depression-like behaviors. In the present study, SIL treatment increased hippocampal TPH-1 mRNA levels, which suggests that TPH-1 mRNA expression and 5-HT signaling played important roles in the anti-depressive activities of this compound. Many studies have reported that the interactions between TPH-1 and the serotonergic system can, in and of themselves, be the basis for the development of antidepressant effects [38]. TPH-1 is responsible for 5-HT synthesis in the brain and may be susceptible to antidepressant activities in vivo [38]. In the present study, there was a significant association between TPH mRNA levels in the brain and immobility time in the FST. The proposed antidepressant effects of TPH are likely regulated by enhanced 5-HT synthesis in the brain [39] because, within the midbrain, 5-HT concentrations are positively correlated with TPH mRNA levels [40].

Therefore, SIL may exert anti-depressant and anxiolytic effects by increasing 5-HT levels and enhancing TPH expression in the Hipp. Based on previous studies, the effective SIL dose (100 mg/kg/day) and an administration period of 2-weeks were selected for the present study. The administration of SIL prevents decreases in TPH expression due to SPS-induced stress and attenuates depressive-like behaviors in rats. Similarly, the present results showed that SIL increased TPH expression and 5-HT concentrations in the brain and also regulated monoamine levels, including those of DA and NE.

## Conclusions

Taken together, the present behavioral findings from the FST and OFT demonstrated that the administration of SIL reduced anxiety- and depression-like behaviors. Furthermore, the results indicated that these behavioral changes likely occurred via regulation of the monoamine system as well as the 5-HT system. Taken together, these findings suggest that SIL may be a useful therapeutic component for the treatment of trauma-associated diseases, including PTSD. As an alternative to FLX, natural products with fewer side effects may also offer the potential for the long-term treatment of anxiety and depression induced by PTSD.

## Data Availability

All data supporting the conclusions of this article are included within the article. The datasets used /or analysed during the current study available from the corresponding author on reasonable request.

## Abbreviations

5-HT: Serotonin
6-OHDA: 6-hydroxydopamine
AD: Alzheimer’s disease
ANOVA: Analysis of variance
CORT: Corticosterone
DA: Dopamine
ELISA: Enzyme-linked immunoassay
FLX: Fluoxetine
FST: Forced swimming test
GAPDH: Glyceraldehyde-3-phosphate dehydrogenase
HPA: Hypothalamic-pituitary-adrenal
LPS: Lipopolysaccharide
MPP: 1-methyl-4-phenylpyridinium
NE: Norepinephrine
NF-κB: Nuclear factor-kappaB
OFT: Open field test
PD: Parkinson’s disease
PTSD: Post-traumatic stress disorder
RT-PCR: Reverse transcription-polymerase chain reaction
SD: Sprague-Dawley
SIL: Silibinin
SPS: Single prolonged stress
SSRIs: Selective serotonin reuptake inhibitors
TPH: Tryptophan hydroxylase
TPH-1: Tryptophan hydroxylase-1
TRP: Tryptophan
